# Unveiling Dermatomyositis: A Tragic Tale of Mortality in a 23-Year-Old

**DOI:** 10.7759/cureus.56058

**Published:** 2024-03-12

**Authors:** Vinit Deolikar, Sarang S Raut, Saket Toshniwal, Shilpa A Gaidhane, Sourya Acharya

**Affiliations:** 1 Internal Medicine, Jawaharlal Nehru Medical College, Wardha, IND

**Keywords:** shawl sign, holster sign, gottron papules, heliotrope sign, s: dermatomyositis

## Abstract

Dermatomyositis represents a rare inflammatory myopathy that induces inflammation in the muscles or related tissues, including the blood vessels supplying these muscles. The precise pathogenesis of this condition remains unknown. Diagnosis typically relies on clinical indicators such as skin rashes, progressive muscle weakness, elevated serum muscle enzymes, abnormal electromyogram results, and muscle biopsy. In this case study, we report a fatal case of dermatomyositis in a 23-year-old female patient who succumbed to complications of dermatomyositis, causing mortality without any evidence of malignancy.

## Introduction

Dermatomyositis is a rare multisystemic disease that predominantly affects the skin, muscles, and blood vessels. This condition typically impacts both adults and children. In adults, dermatomyositis commonly emerges in the late 40s to early 60s. In children, it manifests between the age group of five and 15 and demonstrates a higher occurrence in females than males [1]. Dermatomyositis displays symmetric, proximal greater than distal muscle weakness along with a characteristic rash that includes the heliotrope rash, Gottron papules (raised erythematous rash over knuckles), V-sign, holster sign, shawl sign over the back of the neck and shoulder, nail bed telangiectasias, and subcutaneous calcium deposits [1].

The etiology of dermatomyositis remains elusive, although genetic, immunological, infectious, and environmental factors have been implicated. Various infectious agents, such as coxsackievirus, parvovirus, human T-cell lymphotropic virus type 1 (HTLV-1), *Toxoplasma* species, and *Borrelia* species, have been proposed as potential triggers for dermatomyositis [2]. In the absence of malignancy, patients with dermatomyositis typically exhibit a favorable prognosis, with five-year survival rates ranging from 70% to 93%. Unfavorable prognostic indicators include advanced age, concomitant interstitial lung disease (ILD), cardiac complications, and delayed or insufficient prior treatment [3].

## Case presentation

In the following case, a 23-year-old female presented to the outpatient department of medicine with complaints of bilateral lower limb weakness for two months, difficulty in walking for two to three months, swelling over the face and bilateral upper limbs for one month, bilateral upper limb weakness for one month, and breathlessness for 15 days. The patient also complained of a recent onset of slurring of speech and difficulty in swallowing for eight to 10 days, which was gradually progressive. The patient was alright three months ago, and then she noticed black pigmentation on her forehead, for which she consulted a private dermatologist and was treated accordingly only with corticosteroid for about one and a half months.

In the meantime, over 10-15 days after the onset of pigmentation over the forehead, the patient developed bilateral lower limb weakness in such a way that the patient was not able to get up from a squatting position and required support while getting up from a sitting position and walking but was able to wear and hold her slippers and was able to dorsiflex and plantarflex her foot. The weakness gradually progressed to bilateral upper limbs in around one to one and a half months, so the patient could not do daily activities such as putting on her clothes, combing her hair, and taking morsels in her mouth. Simultaneously with bilateral upper limb weakness, the patient also developed facial swelling, especially around her eyes. The patient also had breathlessness on rest, which was insidious in onset and progressive in nature. With the progression of the disease, the patient was advised to visit a tertiary center for further management.

**Figure 1:**
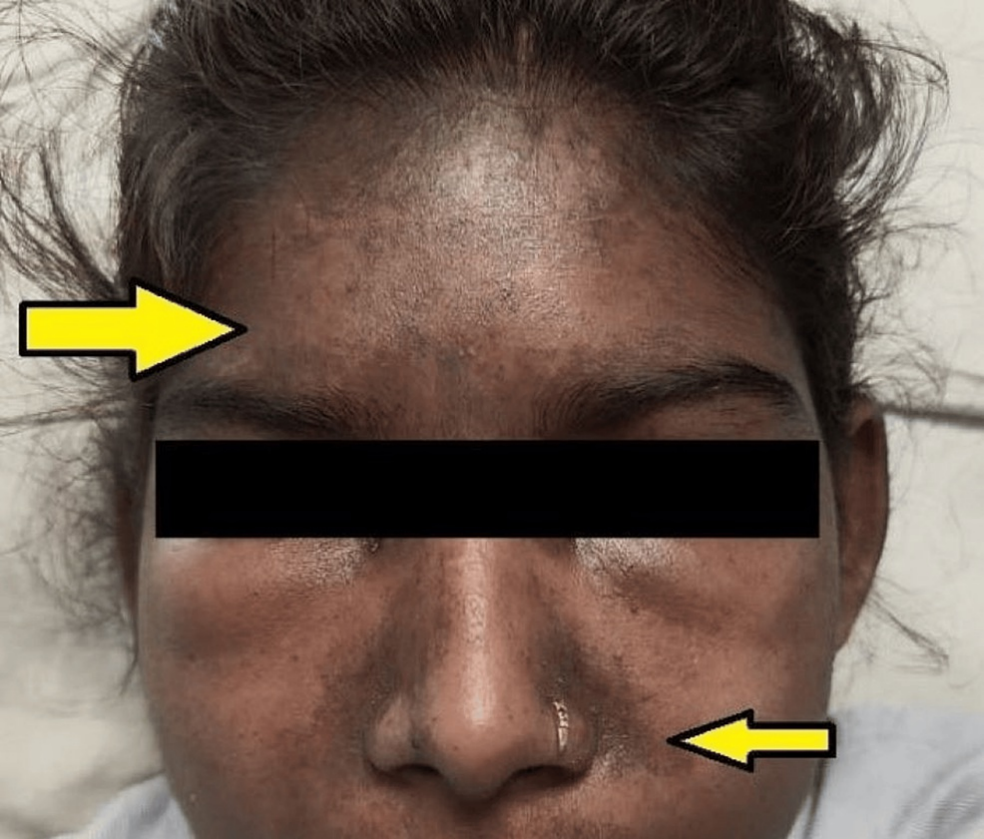
A reddish-purple rash on the upper eyelid, across the cheeks, on the bridge of the nose in a butterfly distribution, and on the forehead and scalp suggestive of a heliotrope rash (as shown by the yellow arrow).

**Figure 2:**
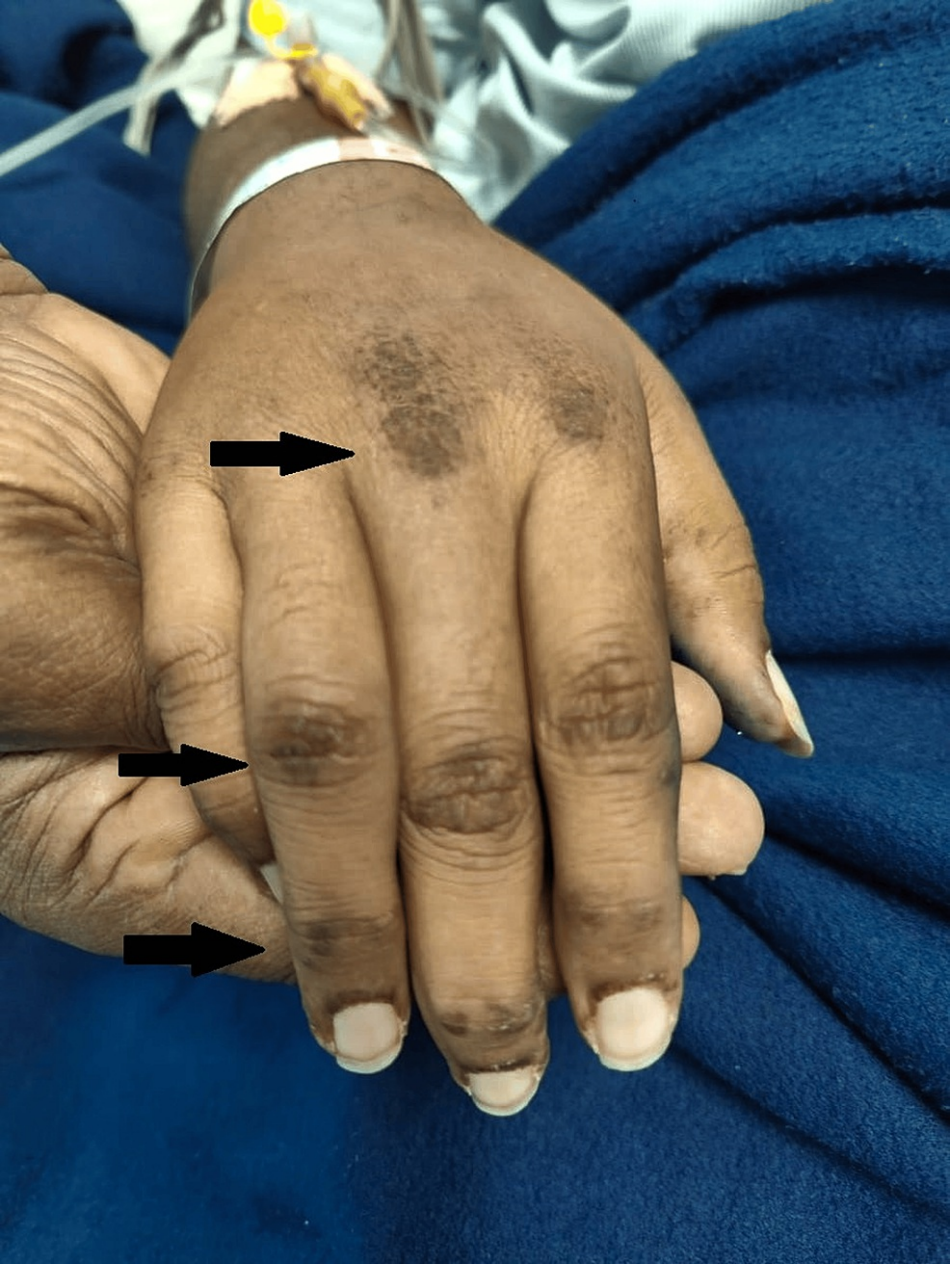
Erythematous to violaceous papules over the extensor surface of metacarpophalangeal and interphalangeal joints suggestive of a Gottron papule (as shown by the black arrow).

**Figure 3:**
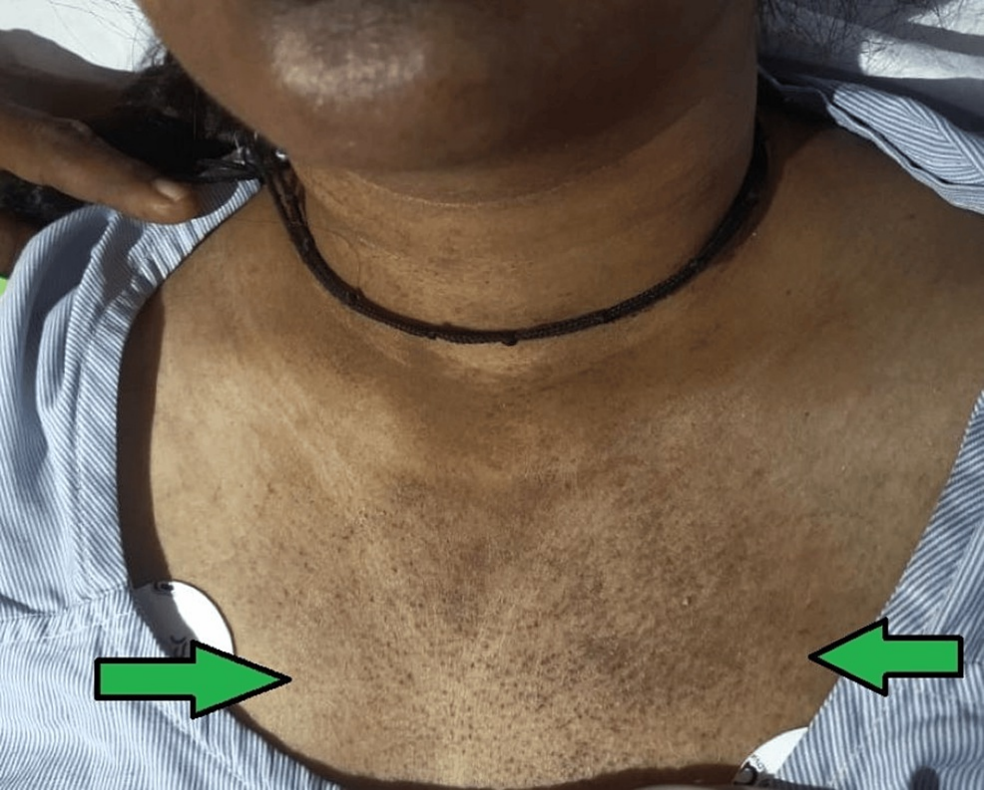
Discrete macular erythema over the sun-exposed part of the anterior neck and upper chest suggestive of a V-neck sign (as shown by the green arrow).

**Figure 4:**
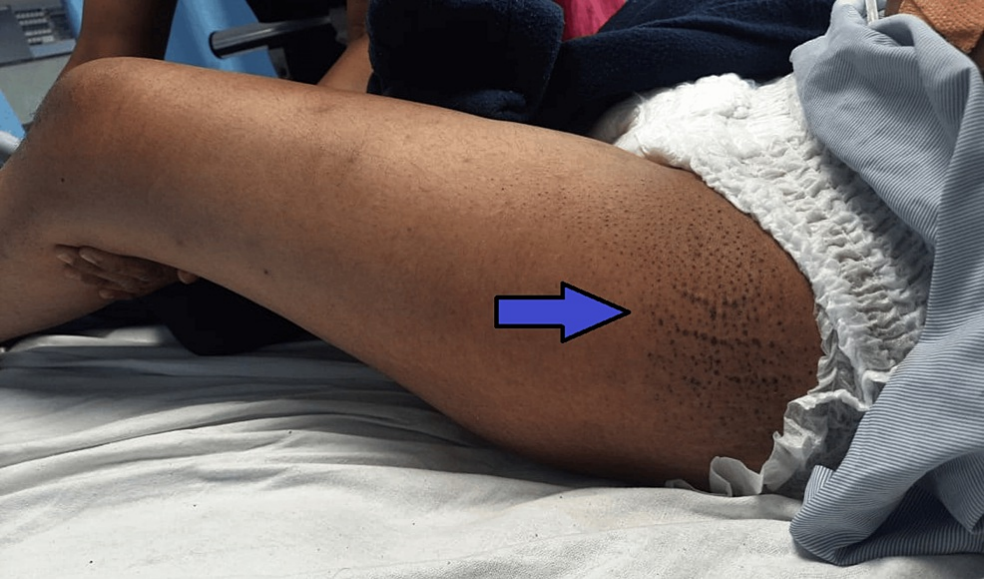
A reddish-purple discoloration on the lateral aspect of the thighs suggestive of a holster sign (as shown by the blue arrow).

Upon comprehensive evaluation, the patient exhibited a normative physique with a body mass index (BMI) of 23 kg/m^2^. The patient manifested normothermia, a pulse rate of 120 beats per minute, a blood pressure of 100/60 mmHg, a respiratory rate of 27 breaths per minute, and an oxygen saturation (SpO2) level of 90% while breathing ambient air. Upon a more detailed examination, the patient displayed erythematous discoloration of the eyelids accompanied by periorbital edema, commonly referred to as heliotrope rash. An erythematous rash was also observed on the extensor surfaces of joints, including the knuckles, elbows, knees, and ankles, indicative of the Gottron sign. Rashes were also present over sun-exposed regions, specifically the anterior neck and chest, forming a V-shaped pattern, commonly known as V-sign. The rashes were also present on the lateral aspect of the thigh, referred to as the holster sign, as shown in Figure 1, Figure 2, Figure 3, and Figure 4. Laboratory blood investigations of the patient are shown in Table 1.

**Table 1:** Laboratory parameters of the patient in reference to the normal range

Lab parameters	Observed value	Normal range
Haemoglobin	10 gm%	13-17 gm%
Mean corpuscular volume	81.5 fL	83-101 fL
Total leucocyte count	8400 cells/mm^3^	4000-10000 cells/mm^3^
Platelets	239,000 mm^3^	150,000-400,000 mm^3^
Urea	21 mg/dL	19-43 mg/dL
Creatinine	1.1 mg/dL	0.66-1.25 mg/dL
Sodium	142 mmol/L	137-145 mmol/L
Potassium	4.2 mmol/L	3.5-5.1 mmol/L
Alkaline phosphatase	438 U/L	38-126 U/L
Alanine transaminase	195 U/L	<50 U/L
Aspartate aminotransferase	652 U/L	17-59 U/L
Albumin	2.7 g/dL	3.5-5 g/dL
Total bilirubin	2.1 mg/dL	0.2-1.3 mg/dL
Conjugated bilirubin	1.5 mg/dL	0-0.3 mg/dL
Unconjugated bilirubin	0.6 mg/dL	0-1.1 mg/dL
Creatine phosphokinase	12800 IU/L	15-130 IU/L
Lactate dehydrogenase	3042 U/L	140-280 U/L

**Figure 5:**
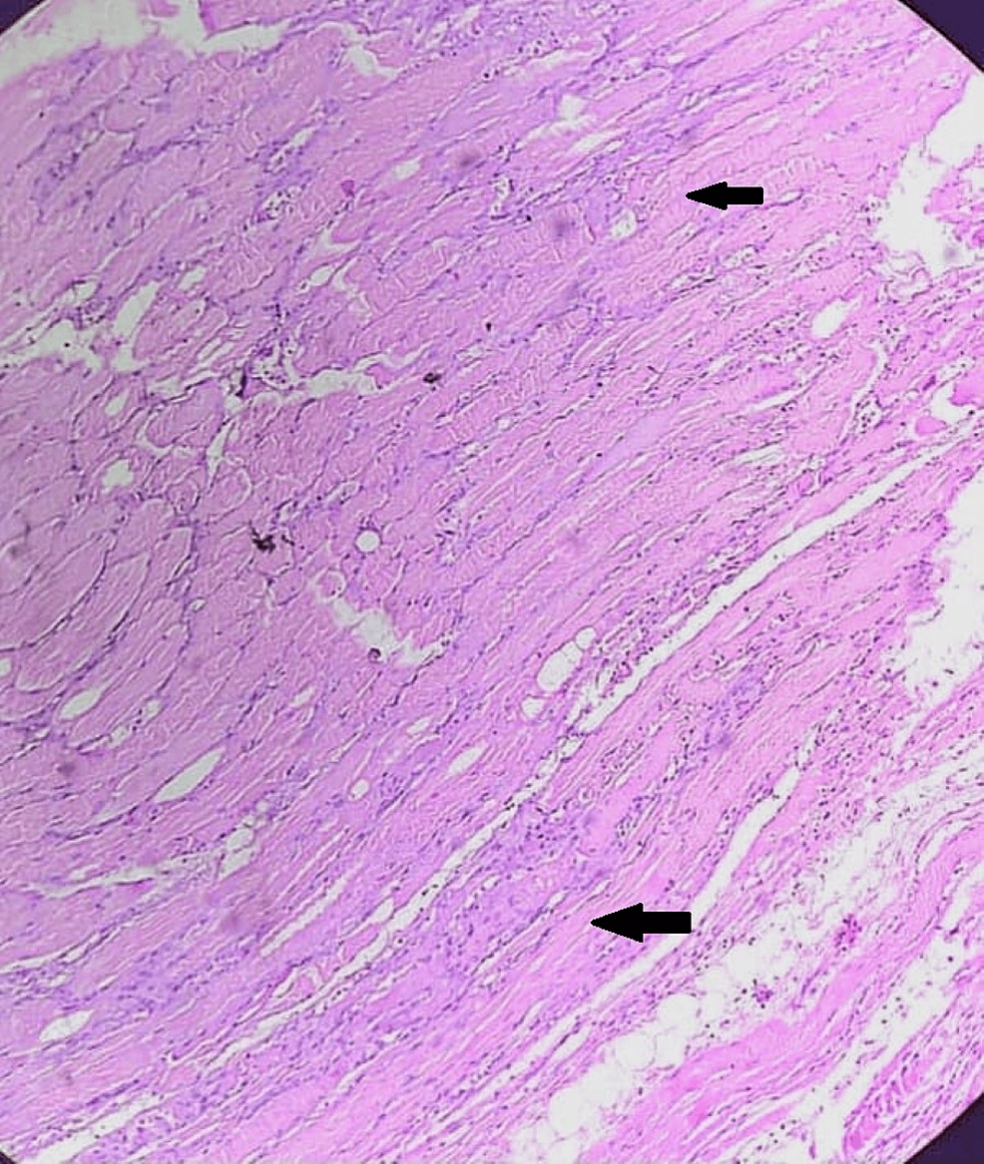
Featured chronic nonspecific inflammatory infiltrates, at places few lymphocytic infiltrates (shown by the black arrow) were seen as suggestive of myositis.

For confirmation of diagnosis, the biopsy was taken from the right thigh, which featured chronic nonspecific inflammatory infiltrates at places where few lymphocytic infiltrates were seen as suggestive of myositis, as shown in Figure 5.

**Figure 6:**
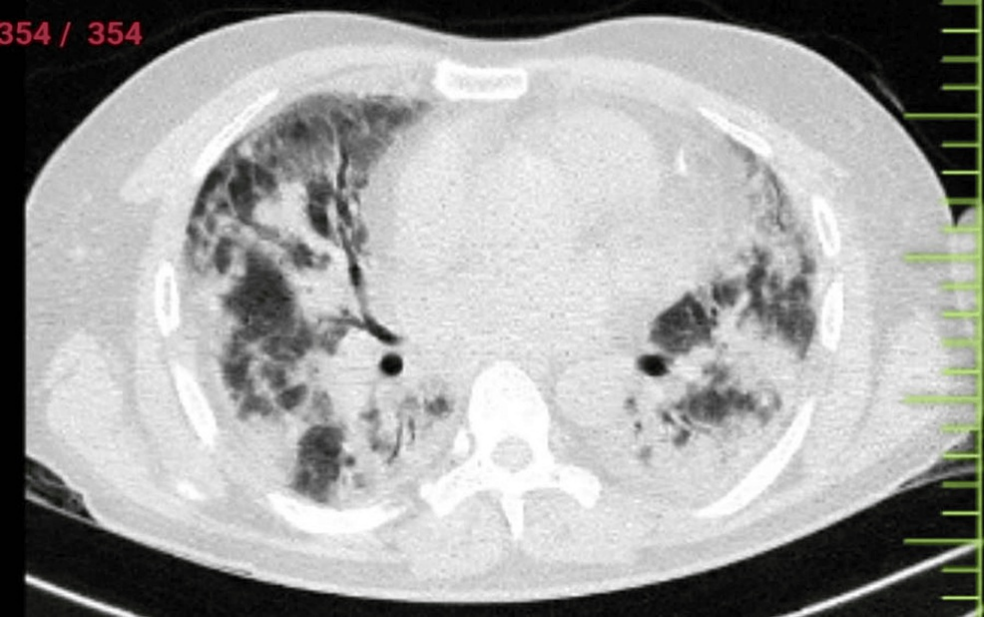
HRCT of the thorax revealing extensive ground-glass opacities with intralobular septal thickening in bilateral lung fields. HRCT: high-resolution computed tomography

Following the confirmation of the biopsy report, the patient commenced intravenous immunoglobulin therapy at a dosage of 2 g/kg based on body weight. Nevertheless, within one week, the patient exhibited respiratory muscle weakness, prompting a transfer to the intensive care unit. Arterial blood gas analysis indicated severe respiratory acidosis. The patient struggled to maintain adequate oxygen saturation, so high-resolution computed tomography (HRCT) was done, suggesting extensive ground-glass opacities with intralobular septal thickening in bilateral lung fields (Figure 6). Mechanical ventilation was initiated due to declining saturation and an elevated respiratory rate. Despite these interventions, the patient's condition continued to deteriorate, ultimately leading to a sudden cardiorespiratory arrest. Despite exhaustive cardiorespiratory resuscitative measures, the patient could not be successfully revived.

## Discussion

Dermatomyositis also impacts other organ systems, such as the pulmonary, cardiovascular, and gastrointestinal systems. What sets dermatomyositis apart is that a notable portion of patients diagnosed with this condition also have an underlying malignancy, which can influence the prognosis and management of the disease. The typical presentation of dermatomyositis involves both muscular and cutaneous symptoms. Nevertheless, there are variations of the condition. Clinically amyopathic dermatomyositis (CADM) is one variant where patients exhibit the characteristic skin findings of dermatomyositis but do not experience muscle weakness. CADM is further categorized into hypomyopathic and amyopathic dermatomyositis. In hypomyopathic dermatomyositis, patients do not display clinical signs of muscle weakness. However, laboratory investigations, electromyography, or muscle biopsy may reveal evidence of myositis (muscle inflammation) [4].

On the other hand, in amyopathic dermatomyositis, patients lack clinical symptoms and laboratory evidence of muscle involvement [4]. This distinction is crucial in guiding diagnosis and management of the condition as it influences treatment approaches and patient care. In our case, the patient had classical dermatological findings of dermatomyositis (heliotrope rash, Gottron papules, V-neck sign, holster sign) along with neurological findings such as bilateral proximal muscle weakness along with muscle tenderness [5,6].

Dermatomyositis may be present with a positive antinuclear antibody but is nonspecific for dermatomyositis. Dermatomyositis is associated with several MSA targeting melanoma differentiating antigen 5 (MDA5), transcriptional intermediary factor 1 (TIFI), and nuclear matrix protein 2 (NXP2). Anti-TIFI and anti-NXP2 are associated with an increased risk of cancer. On the other hand, anti-Mi2 antibodies are associated with a benign form of dermatomyositis and have favorable treatment [7,8].

The pathogenesis of dermatomyositis was traditionally thought to involve an antibody-mediated assault on endothelial cells, leading to complement-mediated capillary destruction and ischemia of muscle fibers. However, recent research indicates otherwise. Immunoglobulin deposition is notably lacking in endothelial cells, and complement deposition might be a secondary occurrence. Mounting evidence suggests that the microvasculopathy, skin, and muscle damage linked to dermatomyositis primarily result from toxicity induced by type I interferon (IFN)-mediated pathways, particularly IFN-β [9,10].

The primary approach in managing dermatomyositis involves the administration of steroids, particularly prednisolone, with an initial dosage ranging from 0.5 to 2 mg/kg/day. High doses of oral prednisone are the preferred choice for early intervention to alleviate muscle weakness. Symptom relief is expected within four weeks, after which a gradual tapering of steroid dosage over 10 weeks is recommended, reaching 1 mg/kg every other day. When prednisone is contraindicated, alternative agents like methotrexate and azathioprine may be considered. In cases of therapy resistance, rituximab, intravenous immunoglobulin, and other biologics prove beneficial. Superficial skin disease can be addressed with antipruritics, topical steroids, hydroxychloroquine, and systemic steroids [11,12].

## Conclusions

Dermatomyositis is a rare multisystemic autoimmune disease that can prove to be lethal if not diagnosed and treated early; in our case, the patient remained undiagnosed and untreated, leading to mortality. Hence, early diagnosis and evaluation of such patients with close observation and classical signs of physical examination, such as periorbital edema with rashes, papules over the extensor surface of minor joints, macular erythema on sun-exposed area, etc., along with neurological features such as proximal muscle weakness and tenderness should be ruled out to avoid misdiagnosis and delay in treatment; it is essential to prevent mortality and to improve the quality of life of the patient. Such rare scenarios should be shed light upon and brought to the literature to increase awareness among practitioners to enhance diagnostic skills and patient management.
